# Soft Wearable Piezoresistive Sensors Based on Natural Rubber Fabricated with a Customized Vat-Based Additive Manufacturing Process

**DOI:** 10.3390/polym15102410

**Published:** 2023-05-22

**Authors:** Antonia Georgopoulou, Sasitorn Srisawadi, Panithi Wiroonpochit, Frank Clemens

**Affiliations:** 1 Department of Advanced Materials and Surfaces, Empa-Swiss Federal Laboratories for Material Science and Technology, Ueberlandstrasse 129, 8600 Dübendorf, Switzerland; 2 National Metal and Materials Technology Center, National Science and Technology Development Agency, 114 Thailand Science Park, Phahonyothin Road, Khlong Nueng, Khlong Luang, Pathum Thani 12120, Thailand; sasitors@mtec.or.th (S.S.); panithiw@mtec.or.th (P.W.)

**Keywords:** natural rubber, additive manufacturing, piezoresistive sensor, wearable electronics, conductive composites, acetylene black

## Abstract

Piezoresistive sensors for monitoring human motions are essential for the prevention and treatment of injury. Natural rubber is a material of renewable origin that can be used for the development of soft wearable sensors. In this study, natural rubber was combined with acetylene black to develop a soft piezoresistive sensing composite for monitoring the motion of human joints. An additive manufacturing technique based on stereolithography was used, and it was seen that the sensors produced with the method could detect even small strains (<10%) successfully. With the same sensor composite fabricated by mold casting, it was not possible to detect low strains reliably. TEM microscopy revealed that the distribution of the filler was not homogeneous for the cast samples, suggesting a directionality of the conductive filler network. For the sensors fabricated through the stereolithography-based method, a homogeneous distribution could be achieved. Based on mechano-electrical characterization, it was seen that the samples produced with AM combined the ability to endure large elongations with a monotonic sensor response. Under dynamic conditions, the sensor response of the samples produced by 3D printing showed lower drift and lower signal relaxation. The piezoresistive sensors were examined for monitoring the motion of the human finger joints. By increasing the bending angle of the sensor, it was possible to increase the sensitivity of the response. With the renewable origin of natural rubber and manufacturing method, the featured sensors can expand the applicability of soft flexible electronics in biomedical applications and devices.

## 1. Introduction

Wearable sensors detect the strain-induced dimensional changes that correspond to physiological and anatomical events happening inside the human body [1,2]. Especially, soft piezoresistive sensors can be used as wearable sensors for health monitoring [3,4,5], prosthetic devices [6] and human–machine interfaces [7]. Soft wearable sensors for motion monitoring have become increasingly popular [8,9]. Especially, for monitoring human joints, the sensors need to be soft and mechanically robust to withstand bending, stretching and pressing under dynamic conditions [10,11]. The sensors should be able to monitor the flexion and extension of the joints, and thus, a monotonic response is highly desirable [12]. These types of sensors are used in patient rehabilitation and the monitoring of athletes for performance enhancement, stress factor estimation and injury prevention [13,14]. Alternatives, such as high-speed cameras, are susceptible to environmental factors and the movement of individuals nearby [15]. Rigid sensors reduce the comfort of the wearable because of the additional weight and limited freedom of movement [16,17]. Thus, key aspects for the development of wearable strain sensors are good sensitivity, large detection range and flexibility [1].

A facile fabrication method for wearable sensors is an additional consideration [18,19]. The most common method to develop wearable sensors is the integration of a functional material into a flexible substrate, such as PDMS [18]. Piezoresistive soft sensors that combine functionality with stretchability have been recently developed without using a substrate [20]. One strategy to produce these soft piezoresistive sensors is to combine an elastomeric material with a conductive filler, such as carbon black [5,21,22]. The resulting composite can exhibit piezoresistive properties and endure large elongations at the same time [23]. Typically, silicone rubber [24,25] and thermoplastic elastomers [26,27,28] are used for the development of piezoresistive sensors. These types of polymers (i.e., synthetic rubber) are based on products of the petroleum industry, the so-called non-renewable resources [29]. As an alternative, natural rubber (NR) is a tree-harvested material that is considered bio-based and eco-friendly [30]. NR has large tensile modulus values, good rigidity and abrasion resistance [5,31,32]. In addition, since it is a product of natural origin, NR is biocompatible and biodegradable [33,34,35].

NR has been combined with conductive fillers for the development of piezoresistive composites [32,36,37,38]. Especially, carbon black is a popular filler for NR composites because it is a common additive in the tire industry to improve mechanical properties. Natarajan et al. produced a piezoresistive sensor based on carbon black and NR, but they could not achieve a monotonic response, since a plateau appeared at low strains [39]. Nakaramontri et al. developed an NR-based sensor with a combination of carbon nanotubes and carbon black [40]. They also noticed a non-monotonic response at low strains in the form of a secondary peak. A similar secondary peak was also observed by He et al. for their NR sensor based on carbon black [41]. To avoid this problem, some researchers have turned to other conductive fillers, such as gold nanowires or ionic liquids [42,43]. The cost of the final product can increase significantly with these additives. There is still the need to develop piezoresistive composites with a monotonic response with cost-efficient functional fillers, such as carbon black.

Additive manufacturing is often used for the prototyping and optimization of small-scale rubber parts and their composites. Particularly, thermoplastic polyurethane has been commercialized as an additive manufacturing material [44,45]. There have been some attempts at exploring additive manufacturing for NR-based materials using vat-based and material jetting 3D printers [46,47,48]. Developing composites that are compatible with additive manufacturing methods will enhance the use of functional NR composites in many fields of stretchable electronics, such as wearable motion sensors [49]. A printable conductive composite of NR was successfully developed using conductive carbon black as filler [50]. Its potential as a piezoresistive strain sensor was demonstrated, but piezoresistive performance, including signal drift and stress relaxation, was not yet investigated.

In this study, the piezoresistive behavior of composites based on NR and acetylene black is investigated. Acetylene black (AB) is a type of carbon black produced with the acetylene exothermic decomposition process that results in high purity [51,52]. As a result, AB has increased crystallization and higher conductivity than other types of carbon black [53,54]. AB has been already used to achieve natural rubber composites with higher thermal conductivity properties [55,56,57,58]. Merzouki et al. have shown that composites with AB show higher thermal conductivity than composites with graphitic carbon black, because a percolating network can be obtained at lower filler concentrations for composites with AB [59]. Vat-based additive manufacturing is used for sensor fabrication. AB has a high surface area and stable chemical properties [51,59,60], which will be an advantage for the current processing method. The aim is to combine an eco-friendly material with a facile and time efficient fabrication process. The comparison between the mechanical and piezoresistive properties of cast and samples produced with the vat-based AM process is presented. The suggested application is the monitoring of human motions, such as the bending of the human finger. Such wearable sensors can have an impact on the applicability of renewable soft matter composites for healthcare devices.

## 2. Materials and Methods

### 2.1. Development of the Composite Material

High-ammonia NR latex was purchased from Natural Art & Technology Co., Ltd. (Rayong, Thailand) with a total solid content (TSC) of about 60%. Acetylene black (AB) was complimentarily supplied by IRPC Public Company Limited (Bangkok, Thailand). NR latex was pre-vulcanized by mixing with sulfur, zinc-2-mercaptobenzothiazole, zinc diethyldithiocarbamate, LOWINOX^®^ CPL stabilizer, zinc oxide and potassium hydroxide. The mixture was stirred for 1 h and kept at 60 °C for 4 h. AB slurry was prepared by mixing cetrimonium bromide (CTAB) solution at 3 wt.% with AB. The slurry was stirred for 1 h before the pre-vulcanized NR latex was added to achieve the AB/NR latex with 7 parts per hundred of rubber (phr) of AB. The AB/NR latex composite was then stirred for 1 h and sonicated for 1 h.

### 2.2. Fabrication Method

The AB/NR latex was formed into thin samples using two different fabrication methods.

#### 2.2.1. Casting

Conventional casting in glass molds with a size of 150 × 150 × 0.5 mm^3^ was used to form conductive AB/NR samples. The composite was dried in the mold for 2 days at room temperature.

#### 2.2.2. Additive Manufacturing

**Figure 1:**
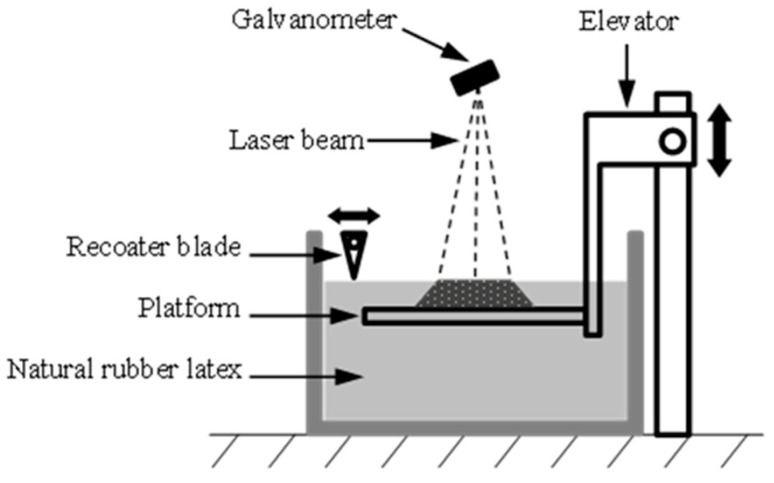
Schematic diagram of the in-house vat-based 3D printer with laser for NR processing.

An in-house developed vat-based 3D printer, shown in Figure 1, is equipped with a low-power nanosecond UV laser (wavelength: 355 nm, average power: 5 W). The design principles are similar to laser-based stereolithography technique except for the absence of a photopolymerization process. The printing process parameters were set as shown in Table 1.

### 2.3. Morphology Characterization

The conductive network of the AB/NR composite was observed using a high-resolution transmission electron microscope (HR-TEM; JEM-2100Plus, JEOL, Tokyo, Japan). The samples were sliced using a Leica Ultracut UCT Ultra-microtome (Leica Microsystems, Germany), and the specimens were placed in a 200-mesh copper grid. Optical microscope images were obtained with the microscope Olympus CX31 HD (Olympus, Tokyo, Japan).

### 2.4. Mechano-Electrical Characterization

For the mechano-electrical characterization, the samples were cut into rectangular sensor strips with dimensions 120 × 5 mm^2^. The testing was carried out on a Zwick & Roell Z005 tensile testing machine (Zwick & Roell GmbH & Co., Ulm, Germany). The electrical signal was recorded with a Keithley 2450 source meter (Keithley Instruments, Solon, OH, USA) by applying a voltage of 20 V. During the tensile testing, the electrical resistance was recorded with a sampling rate of 10 Hz. Tensile testing was performed until the point of fracture. Dynamic properties were investigated using 10 cycles between 0–10%, 50–60% and 0–100% strain. Quasi-static testing was performed for 5 cycles between 0–50% strain by applying a constant strain of 60 s at 0 and 50% strain. For all the tensile measurements, the strain rate was 200 mm/s. The measurements were carried out over three samples. The average between the measurements was plotted.

The relative resistance (*R_rel_*) was calculated using the following formula:

(1) $$R_{rel}=\frac{R-R_{0}}{R_{0}}$$

where *R* is the value of the electrical resistance, and *R*_0_ is the resistance value without applied strain.

The electrical drift was calculated as the percentage difference of the resistance value at maximum strain between the second and tenth cycles of the dynamic testing. The electrical relaxation was calculated as the percentage difference of the relative resistance at the beginning and end of the dwell time [5]. Data were presented as mean values ± standard deviation with sample size (*n* = 3). Statistical analysis was carried out using MS-Excel 2016 Software (Redmond, Washington, DC, USA).

### 2.5. Sensor Device to Demonstrate Human Interactive Wearable Application

**Figure 2:**
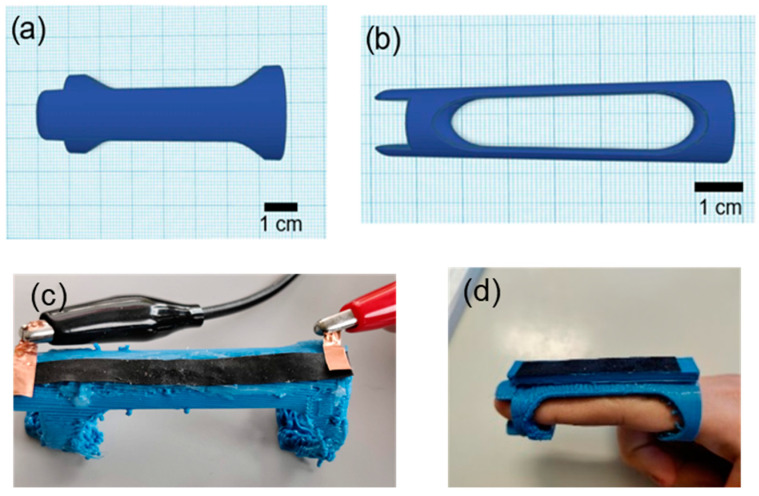
The flexible finger sensor demonstrator used for attaching the sensor strips for testing on a human finger joint (**a**) top view and (**b**) side view. (**c**) Picture of the demonstrator with attached electrodes for recording the electrical signal. (**d**) The demonstrator with a thicker belt on a human finger.

To investigate the applicability of the fabricated sensors in wearable devices, a frame for the AB/NR to attach to the finger (Figure 2) was printed using a thermoplastic polyurethane (TPU) filament with Shore hardness 90A (Spectrum Group, Pecice, Poland) and a filament-based FDM 3D printer Raise3D Pro2 (Raise 3D, Irvine, CA, USA). The sensor strips with dimensions 120 × 5 mm^2^ were attached to the surface of the finger wearable sensor demonstrator using Sil-Poxy silicone glue from Smooth-ON (Macungie, PA, USA). To contact the end of the sensor strips with electrical cables, commercial copper tape (3M, Maplewood, NJ, USA) was used. The demonstrator was tested on a human finger by performing quasi-static testing with a dwell time of 5 s at the bent and extended positions of the finger. The electrical resistance was recorded with the Keithley 2450 source meter with a sampling frequency of 1000 Hz.

### 2.6. Soft Robotic Finger Demonstrator

To monitor the sensor behavior under controlled conditions, a soft robotic bending actuator was fabricated using a thermoplastic polyurethane (TPU) filament with Shore hardness 90A from Spectrum Group and the Raise3D Pro2 3D printer. The frame and actuation have been described in detail in a previous study [43]. A stainless steel tendon wire with 0.5 mm diameter and a Dynamixel AX-12A servomotor from Robotis (Lake Forest, IL, USA) were used to actuate the soft bending actuator. The control was performed with an Arduino microcontroller. The sensor attachment and characterization were performed by dynamic and quasi-static tests, as described in Section 2.5.

## 3. Results

### 3.1. Micrographs of Conductive Networks

**Figure 3:**
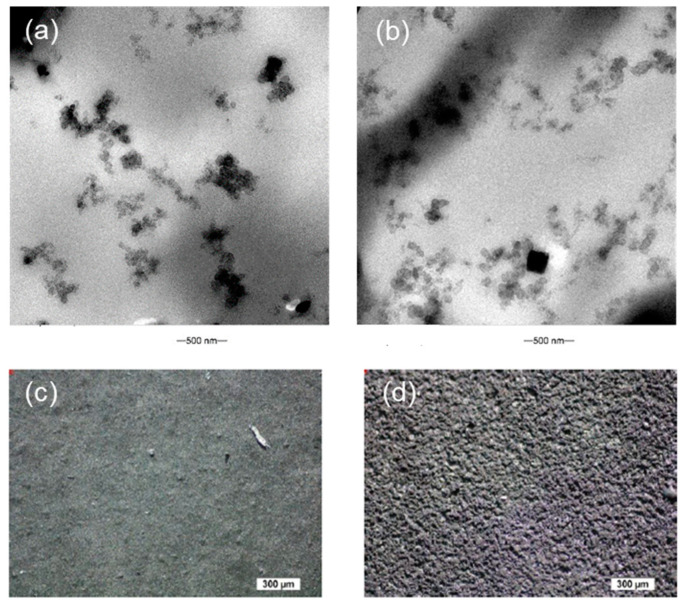
TEM images of the (**a**) cast and (**b**) vat-based 3D printed AB/NR composite strips. Optical microscope pictures of the bottom side of (**c**) cast and (**d**) vat-based 3D printed AB/NR composite strips.

To investigate the connectivity and formation of the AB particle networks, the AB/NR composites fabricated by casting and 3D printing were analyzed by the HR-TEM and shown in Figure 3. The AB particles appear as dark spots that form a network. The network was arranged around spherical rubber particles with particle sizes ranging from 500 to 1000 nm. In Figure 3a, it can be seen that for the casting method, AB tended to agglomerate, which reflected a poor distribution of the AB. In contrast, Figure 3b shows the composites fabricated by the vat-based 3D printer. A more interconnected network without accumulated AB areas was observed. This is in good agreement with the results reported by Srimongkol et al. [50]. The black square-shaped particles in Figure 3b were identified as the zinc oxide (ZnO), which was added to the NR latex during the pre-vulcanization process.

Furthermore, optical microscope pictures were obtained from the bottom side of the cast and 3D printed strips (Figure 3c,d). It was seen that the surface of the 3D printed strips had a higher roughness compared to the strips produced with the casting method. This difference was attributed to the rapid water evaporation during the laser irradiation of the VAT-based 3D printing method.

### 3.2. Tensile Test to the Point of Fracture

**Figure 4:**
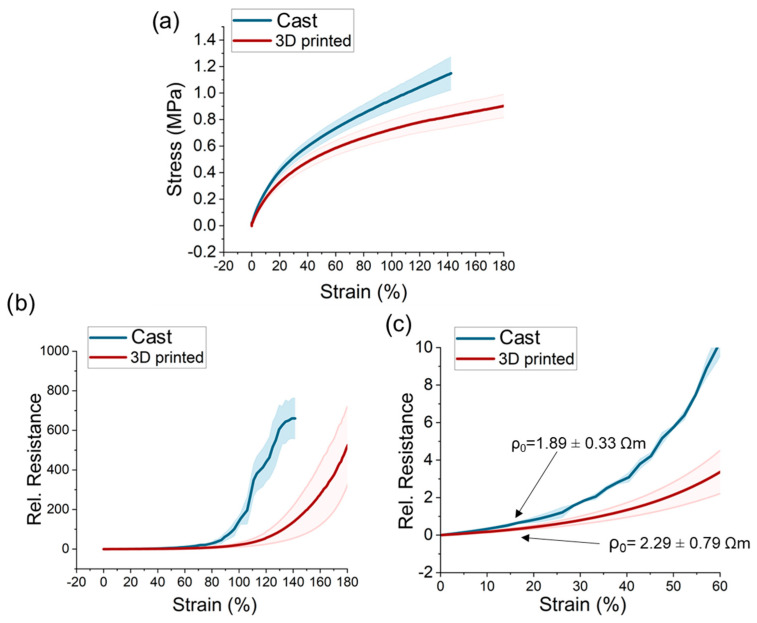
The mechanical and electrical response of the AB/NR composite strips during the tensile test to the point of fracture. (**a**) The stress vs. strain, (**b**) the electrical resistance vs. strain behavior and (**c**) the electrical resistance at low strains.

Cast and vat-based 3D printed sensor strips were investigated by tensile testing experiments to the point of fracture. The mechanical stress and strain of the AB/NR composite strips are shown in Figure 4a. After the shaping process, the cast and 3D-printed samples resulted in a thickness of 0.3 mm and 0.5 mm, respectively. It was observed that the cast samples had overall higher values of stress, and the point of fracture was lower (140% strain). The 3D-printed samples were able to endure elongations up to 180%. The lower strain of fracture of the cast samples is in good agreement with the more inhomogeneous AB filler distribution observed in TEM analysis.

For the electrical response, initial resistivity of 1.89 ± 0.33 Ωm and 2.29 ± 0.79 Ωm could be achieved for cast and 3D-printed sensor strips, respectively. In Figure 4b, it can be seen that both samples exhibited a positive piezoresistive response with the relative resistance increasing sharply above 75% strain. For the cast samples, a sharp increase in the resistance was observed at lower strain. A more inhomogeneous distribution of conductive fillers will result in a faster breakage of the conductive network. This confirms the TEM analysis discussed earlier.

At both high and low strains, the cast sensor strips exhibited higher sensitivity, which was defined as the change in the relative resistance (Δ*R_rel_*). For low strains (Figure 4c), a sensitivity of 8 and 4 could be analyzed for the cast and 3D-printed samples, respectively. Therefore, the cast sample exhibited a two times higher sensitivity. The consistency in quality (standard deviation) was slightly lower for the cast samples.

### 3.3. Dynamic Tensile Testing

**Figure 5:**
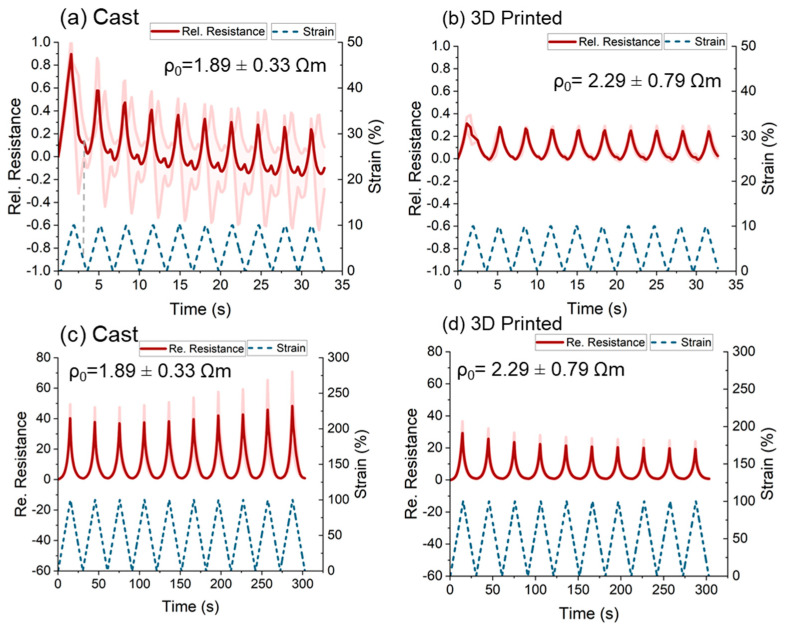
Electrical signal response at a range of strains—0–50% for (**a**) cast and (**b**) 3D-printed sensor strips. Electrical signal response at a range of strains for 0–100% (**c**) cast and (**d**) 3D-printed sensor strips.

A dynamic tensile test of 10 cycles was performed at low (0–10%) and higher strains (0–100%), as shown in Figure 5. At low strains (Figure 5a,b), a positive piezoresistive response occurred for cast and 3D printed sensor strips. The cast sensor strips exhibited larger sensitivity (GF = 9), but a secondary peak appeared at low strains (0.8%) in the unloading. Moreover, the standard deviation was significantly higher compared to the 3D-printed sensor strips. For the 3D-printed sensor strips, the response was monotonic, but the sensitivity was significantly smaller (GF = 3), as expected from the previous tests described in Section 3.2 for the cast sensor strips.

As expected, the relative sensitivity increased significantly for both sensor strips by applying a higher strain (Figure 5c,d). Similar to all previous results, the cast sensor strips showed higher sensitivity. However, the secondary peak in the sensor signal disappeared.

**Figure 6:**
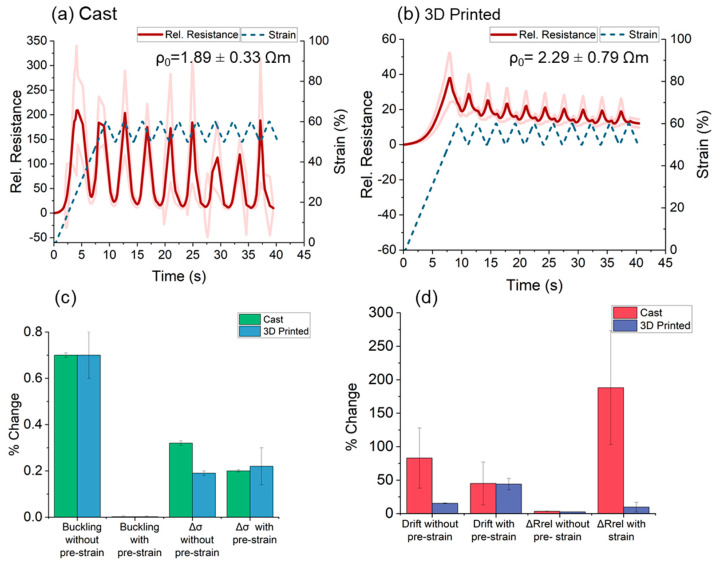
Electrical signal response at a range of strains 50–60% for (**a**) the cast and (**b**) the 3D-printed sensor strips. Summary of the cycling results of 10% elongation with and without pre-straining, (**c**) comparison of mechanical behavior and (**d**) comparison of the electrical behavior of the cast and 3D-printed strips.

In the past, Georgopoulou et al. introduced the strategy of pre-straining as a method for improving aspects of the sensor performance, such as the drift and the linearity [27,61]. Based on the fact that the target was to detect low strains, pre-straining of 50% was used (i.e., 50–60% strain). The sensitivity of the cast samples increased by more than two orders of magnitude (Figure 6a). However, the sensor response was not reproducible between the different cycles (i.e., fluctuation of sensitivity, high drift and secondary peaks), and the standard deviation was high. A similar unreliable response with high drift and secondary peak was observed for the 3D-printed sample (Figure 6b). This unreliable response was attributed to the higher initial resistance (*R*_0_) resulted in the pre-straining to 50% elongation.

For cast and 3D-printed strips, the buckling disappeared due to the pre-straining (Figure 6c). The buckling is an effect linked with the viscoelasticity of the elastomeric matrix and is well known for rubber-based sensors [5]. When buckling is present, the samples do not recover their initial length after straining. This can be identified by the value of the stress becoming zero or negative (Figure S1). Pre-straining in values higher than the buckling appears will help remove the effect [27,61]. This was seen in Figure S2, where the stress was positive in the entire strain range. The value of the maximum stress (strength) was higher for the cast sensor strips at low strains but similar for the two types of sensor strips at higher strains (Figures S1 and S2). This could be a result of the Mullins effect, but there is also an influence of the fabrication process. This observation is in agreement with Figure 4. After pre-straining, the sensitivity (Δ*R_rel_*) increased significantly for the cast samples. However, due to the limitation of the source meter, it was difficult to measure the piezoresistive signal reliably. Based on the deviation, no significant change in the drift value for both sensor strips could be observed (Figure 6d). Overall, the pre-straining strategy was useful for improving the sensitivity, but other aspects of the sensor response, such as a reproducible monotonic response, were impaired. Therefore, the pre-straining strategy was not further equipped in this study.

### 3.4. Quasi-Static Testing

**Figure 7:**
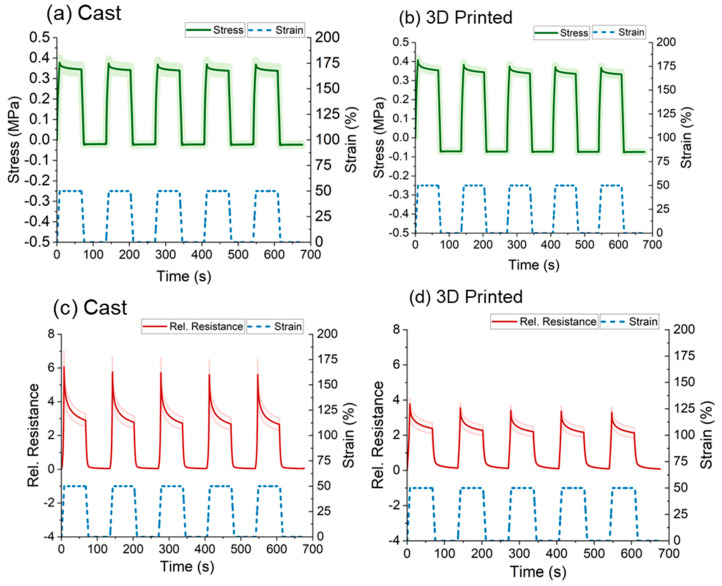
Mechanical response during quasi-static testing at a range of strains 0–50% and for (**a**) the cast and (**b**) 3D-printed sensor strips. Electrical signal response during the quasi-static test for (**c**) cast and (**d**) 3D-printed sensor strips.

Since the response was not monotonic in the range of 0–10% strain, the quasi-static test was performed at 0–50% strain. The fact that the response was monotonic in this range was confirmed with dynamic test (Figure S3). During the quasi-static testing, a dwell time was introduced at maximum and minimum strain. During the dwell time, stress relaxation appeared for both sensor strips. This behavior was expected for elastomer-based composites because of the viscoelastic behavior of elastomers. The value of the stress relaxation (Figure 7a,b) was identical for the cast and the 3D-printed sensor strips (9%).

As for the response of the sensor signal, in the case of the cast sensor strips (Figure 7c), the overshoot at the beginning of the dwell time was more prominent (70% electrical signal relaxation). As shown in Figure 7d, the same value was smaller (43%) for the 3D-printed sensor strips. As expected from the previous results, the relative resistance change was higher for the cast sensor strips.

### 3.5. Application: Wearable Device to Monitor Finger Motion

**Figure 8:**
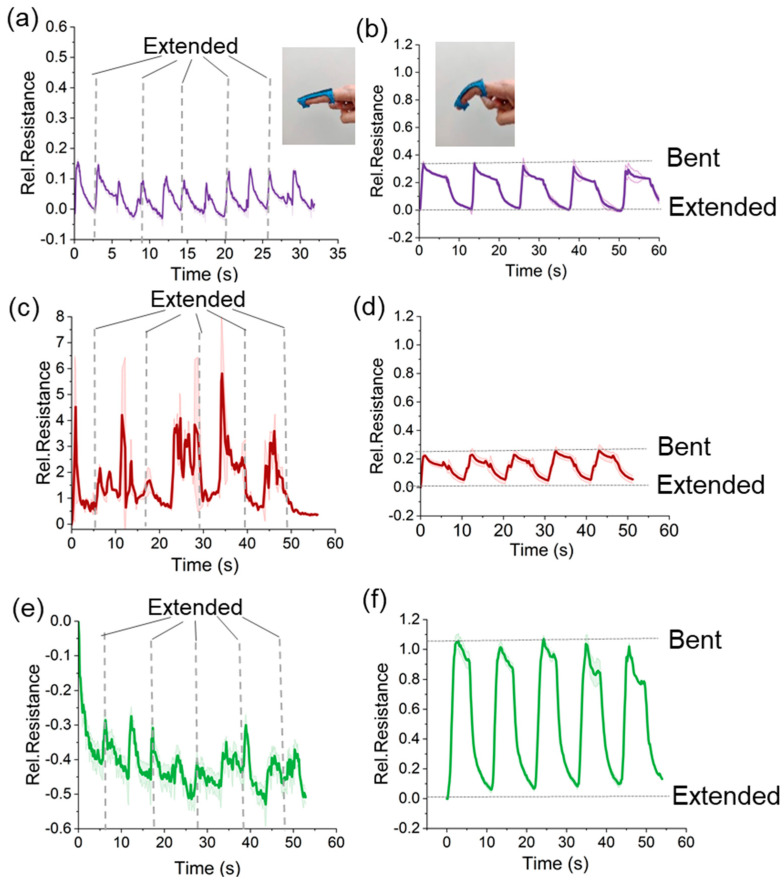
The signal response during bending and extending the finger joint for (**a**) the cast and (**b**) 3D-printed samples attached on a flexible frame for a finger with the electrodes connected on the top of the sensor strips. The same experiment was repeated with the electrodes on the bottom of the sensor strips for (**c**) the cast and (**d**) the 3D-printed samples. In addition, the AB/NR composite strips were mounted on a frame with a spacer of 5 mm. The sensor response for the same experiment is shown for the (**e**) cast and (**f**) 3D-printed sensor strips.

To investigate the applicability of the sensor strips for monitoring the bending of the finger, the sensor strips were fixed on a 3D-printed flexible TPU prototype. The results of the finger signal are reported in Figure 8a,d. The cast sensor strips could not be used for recognizing the position of the finger. The sensor signal of the cast sample increased during the bending but also increased for the extension of the finger. Therefore, it was not possible to distinguish between the bent and extended finger positions. For the 3D printed sensor strips, the finger movement could be detected correctly. There was relaxation present during the dwell time. This behavior was expected based on the quasi-static tensile testing in Figure 7b. Despite the relaxation, the response was repeatable, and it was possible to distinguish between the two finger positions.

For the cast samples, the total thickness (0.5 mm) was achieved in one step, whereas for the 3D-printing, the thickness was achieved layer by layer. To investigate the gradient effect, the AB/NR composite strips were contacted on the bottom side of the samples (Figure 8c,d). In Table 2, the resistivity values of the extended and bent positions are listed for the two different electrode connections on the top and bottom of the sensor strip. Interestingly, in the extended position (i.e., unstrained position), no significant difference between the resistivity for the cast and 3D printed sensor strips could be observed. Nonetheless, in the extended position, a difference in resistivity could be detected for the 3D-printed sensor strips. On the top side, a resistivity of 4.0 Ωm was observed whereas, on the bottom side, the resistance increased to 11.5 Ωm. This difference was significant and could indicate a directionality in the AB networks in the composite.

An option for improving the signal response of the sensor strips, by allocating the straining to higher elongation, would be to increase the bending radius by increasing the distance of the sensor material from the center (neutral bending axis). This was achieved by printing a TPU frame for the finger with a 5 mm spacer. As expected, the higher distance from the neutral bending axis resulted in higher tensile stress and almost five times higher sensitivity (Δ*R_rel_*). Therefore, these results can be used as a guideline for designing wearable devices with the AB/NR composite sensor material to monitor the motion of human joints.

**Figure 9:**
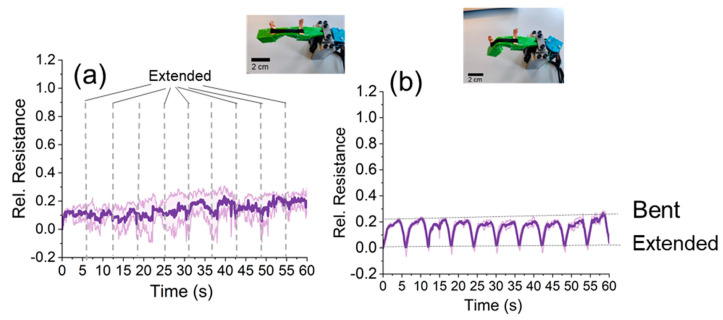
The electrical signal response for the (**a**) cast and (**b**) 3D-printed sensor strips attached to the soft robotic bending actuator.

The bending experiments and the tensile test analysis of the cast sensor strips did not show the expected correlation. Therefore, to avoid the human factor error, bending experiments were performed on a soft robotic bending actuator [43]. The soft robotic bending actuator was driven with a tendon and a servomotor to achieve repeatable and constant bending conditions. The results are shown in Figure 9. Unfortunately, the cast sensor strips could not be used to distinguish between the bent and extension position of the soft robotic bending actuator (Figure 9a). Similar to the finger experiments, it was possible to distinguish between bent and extension positions using the 3D-printed sensor strips (Figure 9b).

## 4. Conclusions

Wearable piezoresistive sensors should combine functionality and the ability to endure large elongations. At the same time, compatibility with facile fabrication methods is highly desired. In this study, piezoresistive sensor composites based on NR and acetylene black were developed. The composites were compatible with a vat-based additive manufacturing method. Cast and 3D-printed sensor strips were compared for their piezoresistive response. The 3D-printed sensor strips could detect low strains (10%) reliably. Below 10% strain, no monotonic signal response of the cast sensor strips could be achieved. Increasing the strain level improved the sensitivity significantly. Testing under dynamic and quasi-static conditions revealed that the 3D-printed sensors showed 5 times lower signal drift and smaller deviation between different samples. In the quasi-static analysis, the signal relaxation during the dwell times was 27% lower for the 3D printed sensors. However, a pre-straining strategy could not be used for neither the cast nor 3D-printed strips. The resistance increased, resulting in a non-reproducible response, where repeating 10 cycles with the same sensor response profile was not possible.

The applicability of the sensor strips for wearable motion monitoring devices was examined by monitoring the motion of the human finger joint. In this case, the cast sensor strips were unsuitable for distinguishing between positions bent and extended of the finger joint. The 3D printed sensor strips could distinguish between the two positions, and the response was reproducible. The same observation was made when testing the sensor strips with a soft robotic finger. A possible interpretation of this difference was attributed to the directionality of the conductive network developed during the fabrication process. This was confirmed by the TEM measurements of the AB network morphology and could be avoided with the 3D-printed sensor strips. These observations will help guide the development of wearable electronic devices with additive manufacturing techniques for future biomedical applications.

## Figures and Tables

**Table 1:** Process parameters for vat-based 3D printer.

Process Parameters	Value
Laser repetition rate (kHz)	70
Scan speed (mm/s)	480
Hatch spacing (µm)	40
Scan pattern	Crosshatching at 0° and 90°
Layer thickness (µm)	150

**Table 2:** The values of the resistivity for each side of the sensor strips during the monitoring of the motion of the finger to bent and extended position.

	Cast	3D-Printed
Resistivity (top) at extended position	2.5 ± 0.1 Ωm	3.8 ± 0.2 Ωm
Resistivity (bottom) at extended position	2.5 ± 0.2 Ωm	3.8 ± 0.2 Ωm
Resistivity (top) at bent position	3.0 ± 0.3 Ωm	4.0 ± 0.1 Ωm
Resistivity (bottom) at bent position	2.9 ± 0.3 Ωm	11.5 ± 1.5 Ωm

## Data Availability

The data will be made available upon reasonable request from the authors.

## Supplementary Materials

The following supporting information can be downloaded at: https://www.mdpi.com/article/10.3390/polym15102410/s1, Figure S1: Dynamic testing 0–10%; Figure S2: Dynamic testing 50–60%; Figure S3: Dynamic testing 0–50%.
