# Supplementary material for: Soft Wearable Piezoresistive Sensors Based on Natural Rubber Fabricated with a Customized Vat-Based Additive Manufacturing Process
Source: Polymers (Basel). 2023 May 22;15(10):2410. doi: 10.3390/polym15102410 (PMC10220999; doi:10.3390/polym15102410)
Supplement: Supplementary file 1 [file polymers-15-02410-s001.zip › polymers-2373951-supplementary.pdf]

Supplementary Figures

Dynamic testing 0-10%

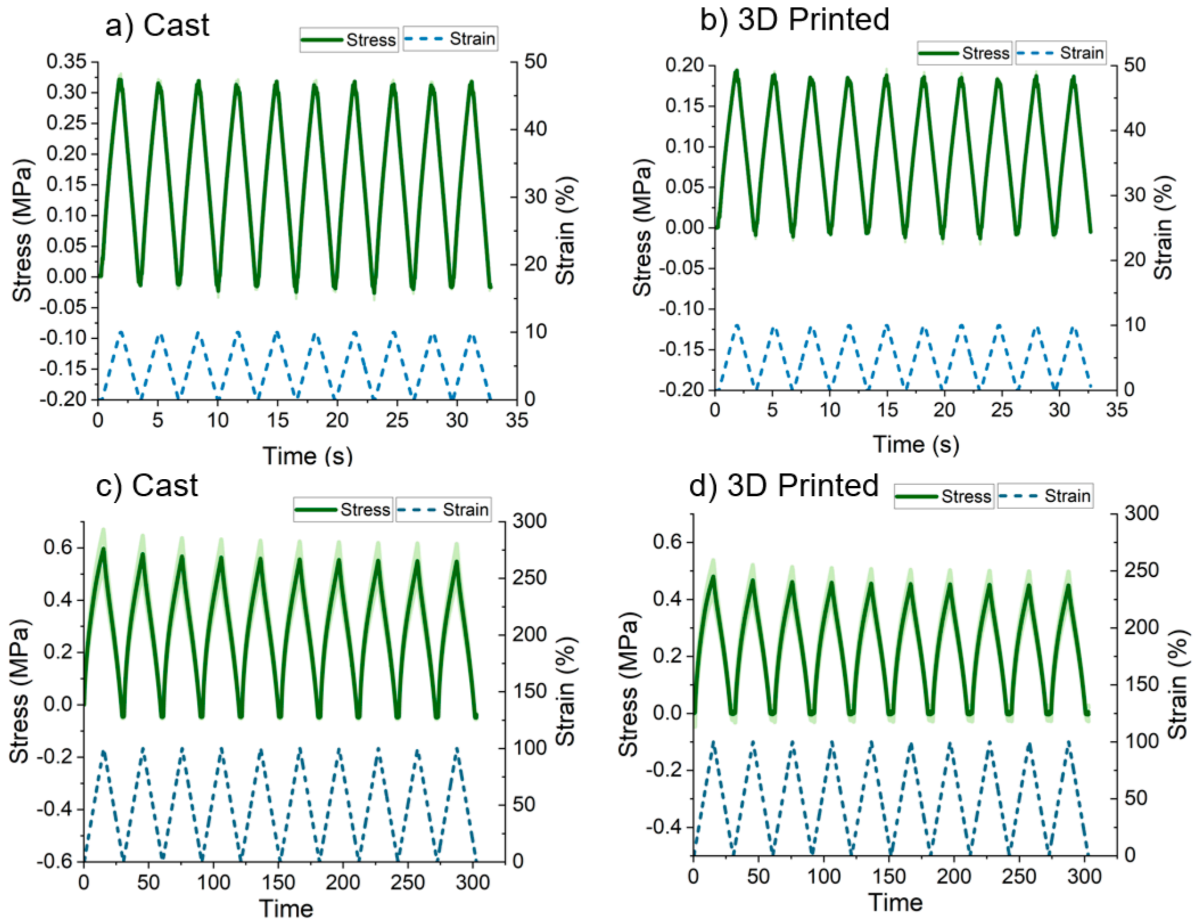

Figure S1: *Response of the mechanical stress during the tensile testing for the cast and 3D printed samples in a range of strains a)-b) 0-10% and c)-d) 0-100%.*

Dynamic testing 50-60%

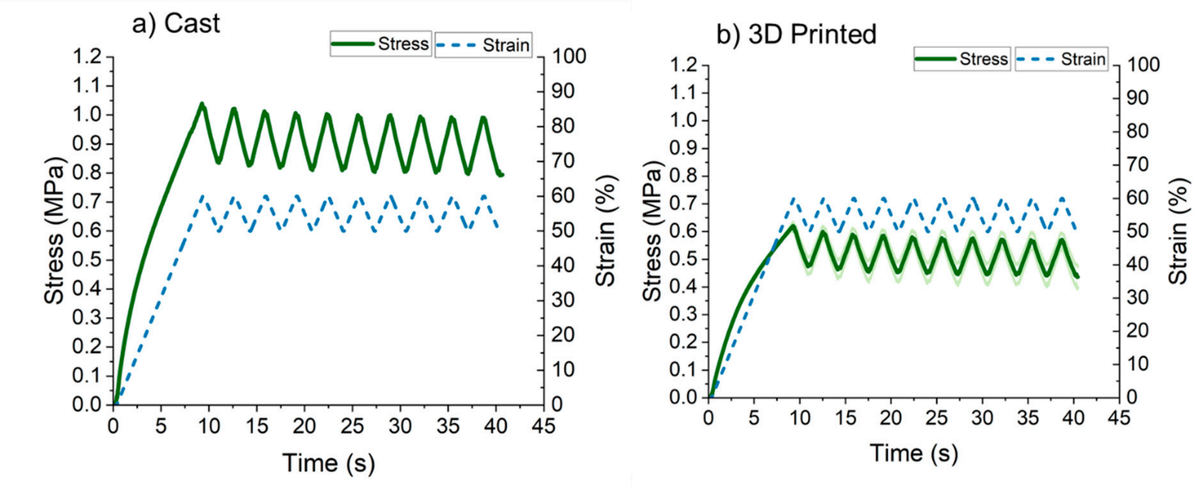

Figure S2: *Response of the mechanical stress during the tensile testing for a) the cast and b) 3D printed samples in a range of strains 50-60%.*

Dynamic testing 0-50%

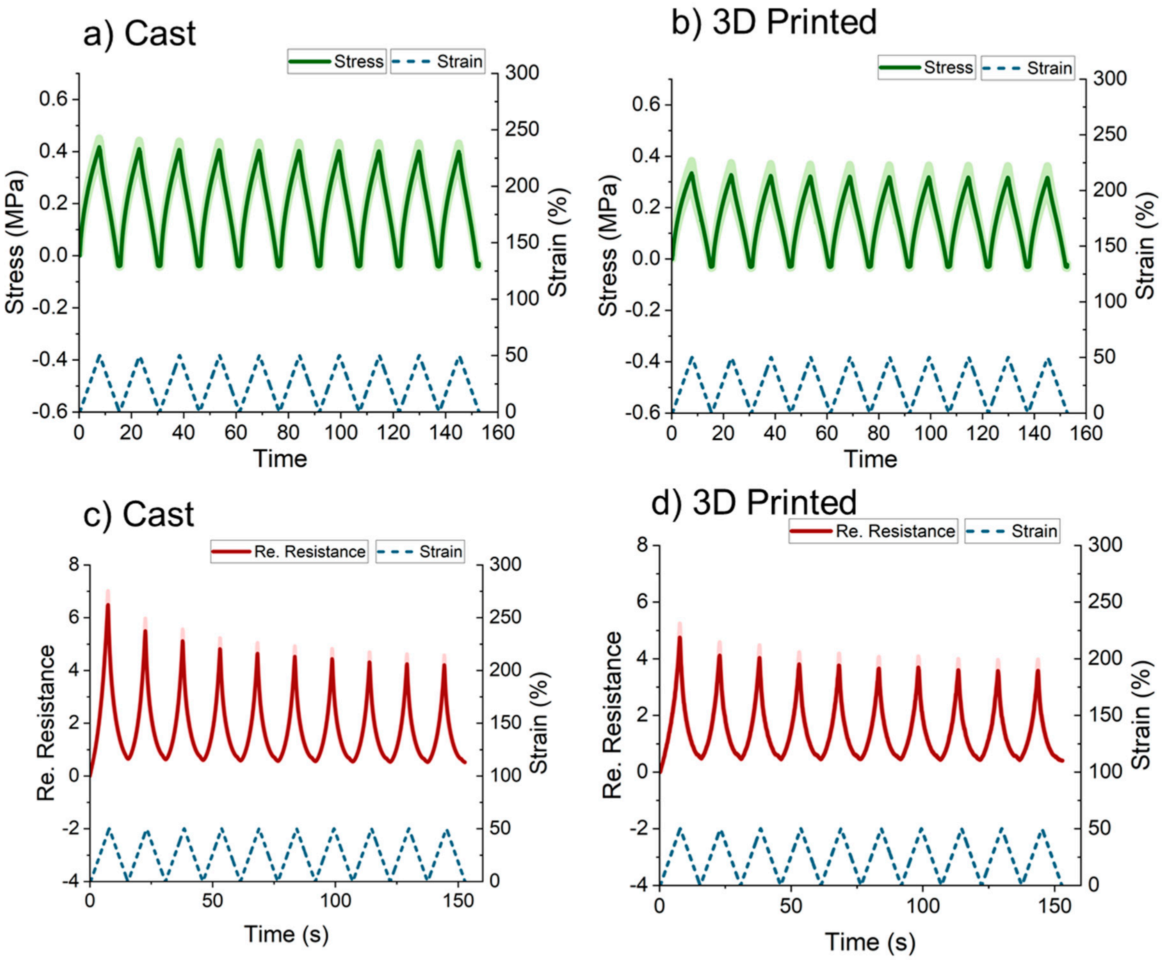

Figure S3: *Response of the mechanical stress during the tensile testing for a) the cast and b) 3D printed samples in a range of strains 0-50%. Response of the relative resistance during the tensile testing for c) the cast and d) 3D printed samples in a range of strains 0-50%.*
